# *Triatoma chiarii* sp. nov. (Hemiptera, Reduviidae, Triatominae): a new species in the *Triatoma brasiliensis* complex from Rio Grande do Norte state, Brazil

**DOI:** 10.1186/s13071-025-07014-4

**Published:** 2025-12-01

**Authors:** Andressa Noronha Barbosa-Silva, Nathan Ravi Medeiros Honorato, Rita de Cássia Moreira de Souza, Carolina Dale, João Luís Reis Cunha, Carlos Eduardo Almeida, Samuel Alexandre Pimenta Carvalho, Ana Carolina Passos, Flávio Campos Ferreira, Paulo Marcos da Matta Guedes, Daniela Maeda Takiya, Liléia Gonçalves Diotaiuti, Daniella Castanheira Bartholomeu, Carlos Ramon do Nascimento Brito, Antonia Claudia Jácome da Câmara, Lúcia Maria da Cunha Galvão

**Affiliations:** 1 Federal University of Rio Grande do Norte, Natal, Rio Grande do Norte Brazil; 2 Federal University of Minas Gerais, Belo Horizonte, Minas Gerais Brazil; 3 Grupo Triatomíneos, Instituto René Rachou, Oswaldo Cruz Foundation (FIOCRUZ) Minas Gerais, Belo Horizonte, Minas Gerais Brazil; 4 Laboratório de Entomologia, Instituto Oswaldo Cruz—Oswaldo Cruz Foundation (IOC—FIOCRUZ), Rio de Janeiro, Rio de Janeiro Brazil; 5 University of York, York, Yorkshire UK; 6 Laboratório Nacional e Internacional de Referência em Taxonomia de Triatomíneos, Instituto Oswaldo Cruz—Oswaldo Cruz Foundation (IOC—FIOCRUZ), Rio de Janeiro, Rio de Janeiro Brazil; 7 Universidade Federal do Rio de Janeiro, Rio de Janeiro, Rio de Janeiro Brazil

**Keywords:** *Triatoma chiarii* sp. nov., Triatomine, *Triatoma brasiliensis* species complex, Chagas disease, Kissing bugs, Geometric morphometrics, Molecular phylogenetics

## Abstract

**Background:**

A new triatomine species was discovered in the semiarid Caatinga region of Rio Grande do Norte, Brazil, where it coexists with *Triatoma brasiliensis* in both natural and artificial habitats.

**Methods:**

Triatomine specimens were captured in peridomestic and sylvatic environments in Rio Grande do Norte. Their identification was based on a combination of analyses, including morphology using dichotomous keys, head and hemelytron morphometry, and phylogeny using cytochrome b and internal transcribed spacer markers.

**Results:**

The new species exhibits morphological traits that are intermediate between those of *Triatoma brasiliensis* and *Triatoma petrocchiae*, but has distinct characteristics, leading to its designation as *Triatoma chiarii* sp. nov. Geometric morphometric analysis of its wings and head clearly distinguished *T. chiarii* sp. nov. from *T. brasiliensis* and *T. petrocchiae*, while phylogenetic reconstruction confirmed its placement within the *T. brasiliensis* species complex. Both approaches consistently supported *T. chiarii* sp. nov. as a species closely related to *T. petrocchiae*, but with sufficient phenotypic and genotypic divergence to warrant its recognition as a new taxonomic entity.

**Conclusions:**

Since *T. chiarii* sp. nov. was also found in the peridomestic environment, its possible role in the eco-epidemiology of Chagas disease warrants further investigation.

**Supplementary Information:**

The online version contains supplementary material available at 10.1186/s13071-025-07014-4.

## Background

Systematic efforts in Brazil to control domiciliated Chagas disease vectors were conducted between 1975 and 1999. During this period, *Triatoma infestans* (Klug, 1834) was the primary vector of Chagas disease in most Brazilian states. Systematic chemical control and housing improvements led to Brazil being certified as free of *T. infestans* vectorial transmission of Chagas disease by 2006 [1, 2]. However, Chagas disease control remains a public health concern in various regions, as autochthonous triatomine species continue to invade and colonize domestic environments, exposing both humans and domestic animals to the risk of *Trypanosoma cruzi* infection [3]. Furthermore, Chagas disease transmission control faces additional challenges, particularly due to oral transmission, which primarily results from consuming açai berries, juices, and other food products contaminated with *T. cruzi* [4–6].

*Triatoma brasiliensis* Neiva, 1911 is the primary vector of Chagas disease in the Caatinga biome, which is located in the semiarid region of northeastern Brazil [7, 8]. This species is the nominal member of the *Triatoma brasiliensis* species complex [9, 10], a monophyletic group that includes the subspecies *Triatoma brasiliensis brasiliensis* and *Triatoma brasiliensis macromelasoma* Galvão, 1956, as well as the species *Triatoma bahiensis* Sherlock and Serafim, 1967; *Triatoma juazeirensis* Costa and Felix, 2007; *Triatoma lenti* Sherlock and Serafim, 1967; *Triatoma melanica* Costa, Argolo and Felix, 2006; *Triatoma petrocchiae* Pinto and Barreto, 1925; and *Triatoma sherlocki* Papa et al., 2002. These taxa exhibit variations in color patterns and morphology [11–13], as well as differences in epidemiological significance, natural history, ecological requirements, and dispersal abilities [8, 14–20].

Specimens of the genus *Triatoma* Laporte, 1832, which did not conform to the typical morphological characteristics outlined in established dichotomous keys [10, 11], were collected from wild and peridomestic environments in 2016 and 2017 in the state of Rio Grande do Norte, Brazil. These specimens exhibited morphological traits that were intermediate between those of *T. brasiliensis* and *T. petrocchiae*. Consequently, we describe *Triatoma chiarii* sp. nov., a new species within the genus *Triatoma*. We additionally conducted morphometric and phylogenetic analyses to assess the morphological and evolutionary relationships of *T. chiarii* sp. nov. in comparison to those of representatives of other species complexes.

## Methods

### Sampling

**Fig. 1:**
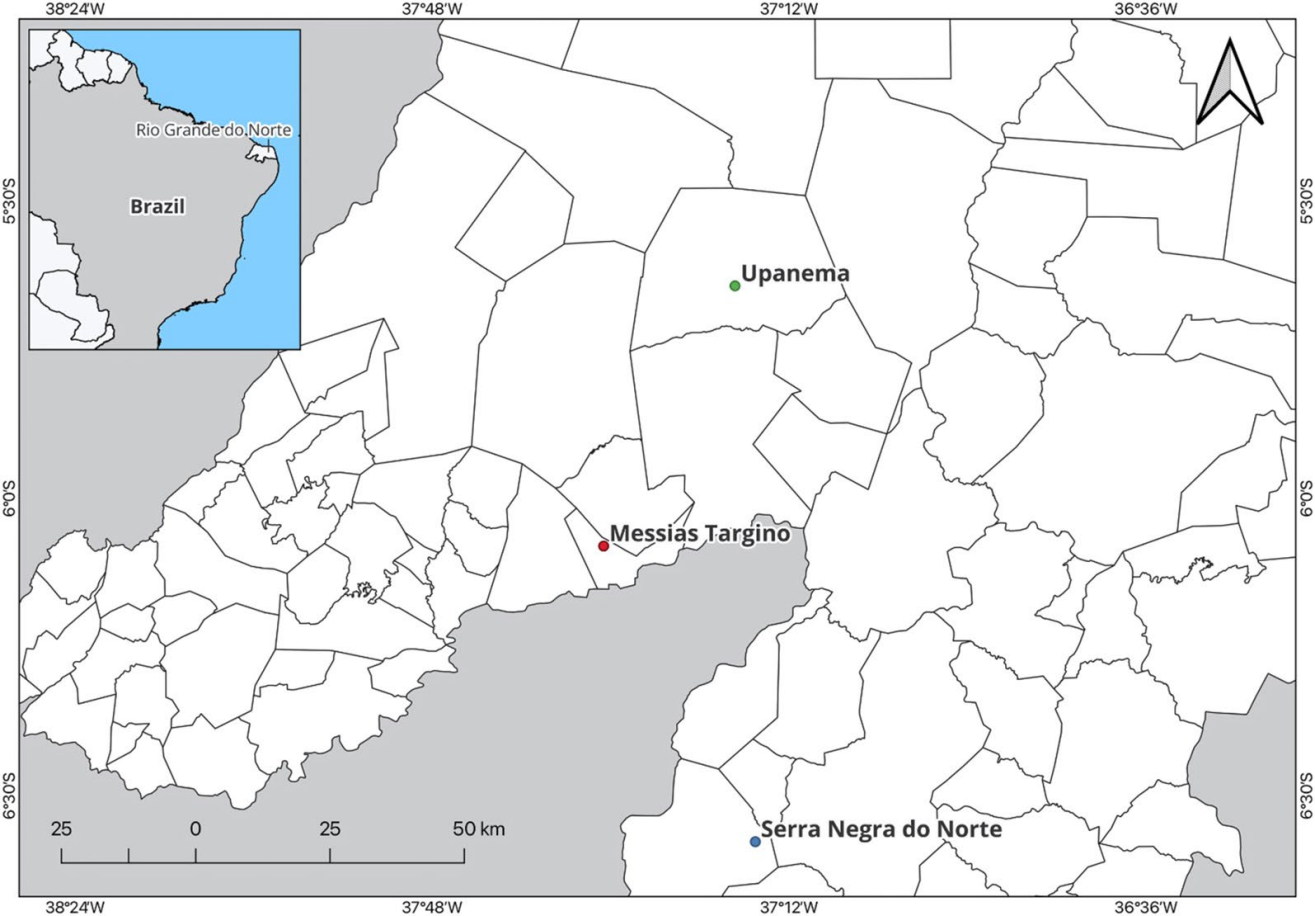
Map showing the exact locations (points) where the triatomine specimens analyzed in this study were collected, in the municipalities of Messias Targino, Upanema, and Serra Negra do Norte in the state of Rio Grande do Norte, Brazil

Eleven adult specimens of *T. chiarii* sp. nov. (four females and six males) were collected from rock outcrops in the sylvatic environment at the Seridó Ecological Station (06°34′37"S, 37°15′24"W), a federal conservation unit managed by the Chico Mendes Institute for Biodiversity Conservation, located in the municipality of Serra Negra do Norte, Rio Grande do Norte, Brazil. Additionally, one adult male specimen was collected from a stack of tiles located in a peridomestic environment, in the municipality of Messias Targino (06°04′47″S, 37°30′42″W), and another in Upanema (05°38′32″S, 37°17′27″W), where they co-occurred with *T. brasiliensis* (Fig. 1). The insects were manually captured in 2016 and 2017 using tweezers and flashlights, without the use of dislodging substances. The insects were individually stored in sealed plastic containers and maintained at – 20 °C until further analysis.

We used dichotomous keys [10, 11, 21] to examine morphological characters, and compared the collected specimens with those of the *T. brasiliensis* species complex deposited in the Trypanosomatid Vector Collection of the Oswaldo Cruz Foundation (FIOCRUZ-COLVET) at the René Rachou Institute, FIOCRUZ Minas Gerais, and with *T. petrocchiae* specimens from the Entomological Collection of the Oswaldo Cruz Institute (FIOCRUZ-CEIOC), Rio de Janeiro.

### Hemelytra and head morphometry

#### Image acquisition and geometric morphometric analysis

The hemelytra (left wing) and the heads (dorsal view) of specimens belonging to species in the *T. brasiliensis* complex were photographed: *T. b. brasiliensis* (10 specimens, five males and five females); *T. b. macromelasoma* (10 specimens, five males and five females; *T. melanica* (eight specimens, seven males and three females); *T. juazeirensis* (10 specimens, five males and five females; *T. lenti* (10 specimens, five males and five females); *T. petrocchiae* (10 specimens, five males and five females); *T. sherlocki* (10 specimens, five males and five females); and *T. chiarii* sp. nov. (16 specimens, nine males and seven females). Images were obtained using a Leica Automontage Magnifier (DMC 2900). Pinned specimens were stabilized with modeling clay, and their wings were kept intact.

**Fig. 2:**
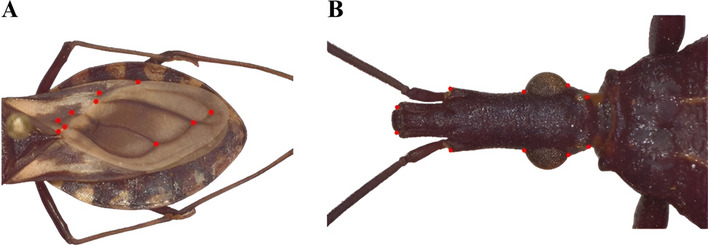
Landmarks on the **A** hemelytron and **B** head used for the morphometric analysis. (Photographs by CD)

Next, 10 type I landmarks were selected on both the hemelytra and heads (Fig. 2A, B) of each specimen [22] by utilizing previously acquired images and TPSdig software version 2.31 [23]. All landmarks were defined at points where distinct structural features converge [24].

#### Data transformation and multivariate analysis

Data were converted into numerical coordinates and organized into a weighted matrix in NTS format using TPSrelw software version 1.75 [23]. After generating the matrix, the centroid size and the uniform* x* and y components were calculated for each specimen, following the methodology described by Coutinho [25] and Passos et al. [26]. The landmark coordinates were then subjected to Procrustes superimposition [24], followed by thin plate spline transformation and discriminant analysis. Multivariate analyses and factorial maps were subsequently generated using JMP software version 17 [27].

Principal component analysis (PCA) and canonical correlation analysis were performed using morphometric data from the hemelytra and heads to determine the differences between the evaluated species. Procrustes coordinate analysis and multivariate ANOVA were subsequently conducted to assess the shape variability. These data were used to generate factorial maps and dendrograms, employing Mahalanobis distances for clustering analysis. Finally, statistical tests (Wilks’ lambda, Pillai’s trace, Hotelling-Lawley trace, and Roy’s max root) were performed for both hemelytra and head analyses using JMP software version 17 [27].

### Molecular analysis

#### DNA extraction and sequencing

DNA was extracted from two legs of each of the 14 triatomine specimens, including seven *Triatoma chiarii* sp. nov., three *Triatoma brasiliensis*, one *Triatoma juazeirensis*, one *Triatoma melanica*, one *Triatoma petrocchiae*, and one *Triatoma sordida*, using the Wizard® Genomic DNA Purification Kit (Promega, Madison, WI), following the manufacturer’s instructions. DNA quantification was performed using a NanoDrop™ 2000 spectrophotometer (Thermo Scientific®, Waltham, MA). The cytochrome b (*cytb*) and internal transcribed spacer-1 (ITS-1) regions were amplified using specific primers (*cytb* [28] and [29]; ITS-1 [30]), following the amplification conditions recommended by the respective authors. PCR products were purified and sequenced using an ABI 3730XL automated sequencer (Applied Biosystems) at the René Rachou Institute–FIOCRUZ Minas Gerais. Sequences from 14 individuals were newly generated in this study, manually checked for quality, and deposited in GenBank (Additional file 1: Table S1).

#### Phylogenetic analysis

*Cytb* and ITS-1 sequences generated herein were aligned with other sequences from GenBank (Additional Table S1). Alignments were conducted using the Geneious algorithm in Geneious Prime for *cytb* [428 base pairs (bp)], and the E-INS algorithm in MAFFT [31] for ITS-1 (1019 bp). Phylogenetic inference was conducted using a concatenated dataset of the two markers under two approaches: maximum likelihood (ML) analysis was performed in IQ-TREE 2.4.0 [32] and Bayesian inference (BI) was conducted in MrBayes 3.2 [33], using two independent runs of four Markov chain Monte Carlo chains for 10 million generations, sampling every 10,000 trees. The model selection and partitioning strategy for the ML analysis were determined by initially partitioning the data by gene and codon positions of *cytb,* and by gene only for the BI analysis, with the restriction on the number of models in ModelFinder [34] in IQ-TREE. Models and partitions selected by BIC were TIMe + G4 for *cytb*_pos1, F81 + F for *cytb*_pos2, TIM3 + F + R2 for *cytb*_pos3, and HKY + F + G4 for ITS-1 in the ML analysis; and GTR + F + I + G4 for *cytb*, and HKY + F + G4 for ITS-1 in the BI analysis. Convergence of independent BI runs was assessed by examining the SD of split frequencies, aiming for a value below 0.05 at the final generation. Additionally, a visual inspection of the sampled parameter distributions was performed using Tracer version 1.7.1 [35]. An effective sample size > 200 was considered the threshold to ensure proper mixing and reliable parameter estimates.

Clade support was assessed by Bayesian posterior probabilities (BPP) and 1000 replicates of the Shimodaira-Hasegawa approximate likelihood ratio test (SH-aLRT; [36]) and 1000 pseudoreplicates of Ultrafast Bootstrap (UFBoot; [37]) in ML.

In addition, we calculated Kimura two-parameter (K2P) distances in MEGA5 [38] to complement the phylogenetic analysis and facilitate comparisons with previous studies on triatomine speciation. The *cytb* gene was selected as a standard marker based on prior literature [29, 39].

## Results

### Taxonomy

Order Hemiptera Linnaeus, 1758

Family Reduviidae Latreille, 1807

Subfamily Triatominae Jeannel, 1919

Genus* Triatoma* Laporte, 1832

*Triatoma chiarii* sp. nov.

### Type material

Holotype female, BRAZIL, Rio Grande do Norte, Serra Negra do Norte, Seridó Ecological Station, coordinates 06°34′37"S, 37°15′24"W. 2016. Wild environment. Number 13341, deposited in FIOCRUZ-COLVET of the regional unit of FIOCRUZ Minas Gerais. Four paratype females, same data as holotype except collection codes, 13339, 13342, 13345, 13348. Six paratype males, same data as holotype except for collection codes 13338, 13340, 13343, 13344, 13346, 13347. One paratype male, Rio Grande do Norte, Upanema, coordinates 05°38′32″ S, 37°17′27″W. 2016. Peridomestic environment 13211, deposited in FIOCRUZ-COLVET. One paratype male, Rio Grande do Norte, Messias Targino, coordinates 06°04′47″S, 37°30′42″W. Peridomestic environment. Fifth November 2017, 10021, deposited in FIOCRUZ-COLVET.

### Type locality

Seridó Ecological Station, Serra Negra do Norte, Rio Grande do Norte State, coordinates 06°34′37"S, 37°15′24"W; Upanema, Rio Grande do Norte, coordinates 05°38′32″S, 37°17′27″W; and Messias Targino, Rio Grande do Norte, 06°04′47″S, 37°30′42″W (Fig. 1).

### ZooBank registration

To comply with the regulations set out in article 8.5 of the amended 2012 version of the International Code of Zoological Nomenclature, details of the new species have been submitted to ZooBank. The Life Science Identifier (LSID) of the article is urn:lsid:zoobank.org:pub:56EFEA36-AC0D-42B5-A83B-A73FDFA06781. The LSID for the new name *Triatoma chiarii* sp. nov. is urn:lsid:zoobank.org:act:49976E40-C8A6-4683-AF61-5D5F3A0AB8ED.

### Etymology

The specific epithet “chiarii” was chosen to honor the distinguished career of Prof. Dr. Egler Chiari (1934–2020), who was internationally recognized for his contributions to the understanding of *T. cruzi*.

### Bionomics

Most of the specimens were found in rock outcrops within the semiarid Caatinga biome. Some adults were found in peridomestic environments without signs of domiciliation.

### Biological observations

Most specimens were found in rock outcrops, but some adults were collected in peridomestic environments, with no evidence of domiciliation. Two specimens collected prior to the formal description were tested for *T. cruzi* infection using optical microscopy of fecal samples and were found to be negative. However, the type series was not screened, as the intention was to preserve specimen integrity.

### Description

**Fig. 3A, B:**
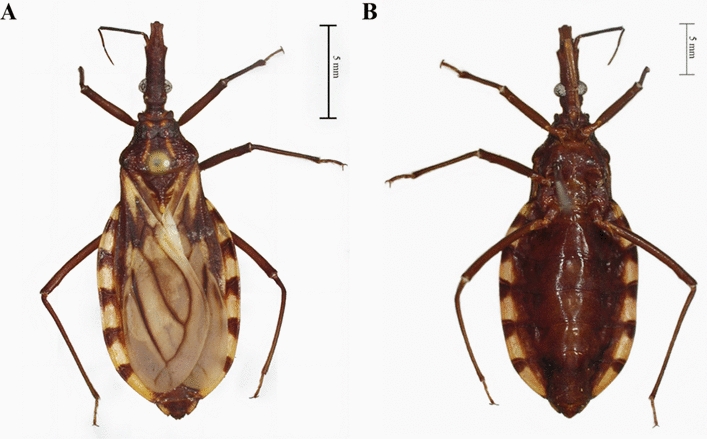
Detail of the female holotype of *Triatoma chiarii* sp. nov. **A** Dorsal view, **B** ventral view. (Photographs by CD)

**Fig. 4A–F:**
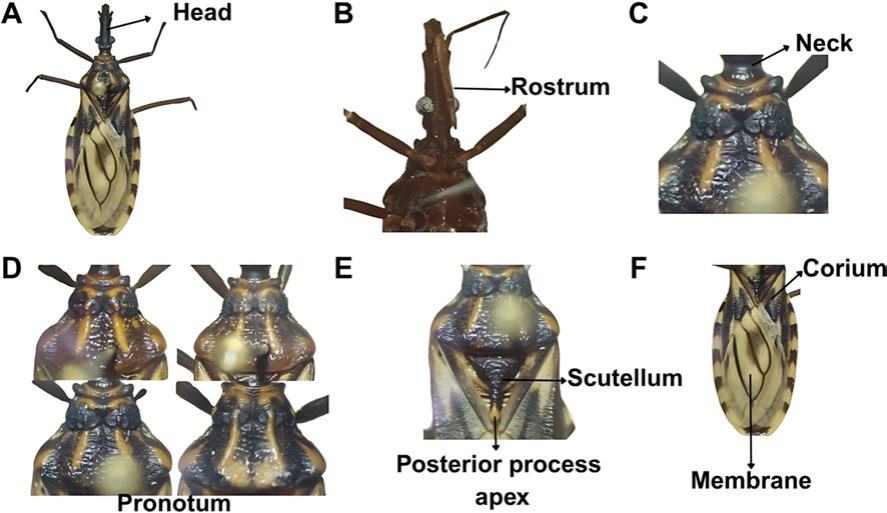
Detail of the male of *Triatoma chiarii* sp. nov. **A** Head, **B** rostrum, **C** neck, **D** pronotum, **E** scutellum, **F** hemelytra. (Photographs by CD)

Length of male 19.0–21.5 mm, of female 19–24 mm; pronotum width of male and female 4.0–5.0 mm. Overall color dark brown, with orange markings on pronotum, and yellowish on scutellum, hemelytra, and connexivum (Fig. 3A, B). Integument appearing glabrous, only with very sparse, short, inapparent setae. Head uniformly dark (Fig. 4A), slightly rugose and granulose, somewhat twice as long as wide across eyes (1:0.40–0.50) and longer than pronotum (1:0.75–0.80). Anteocular region 6 times as long as postocular (1:0.16–0.17), postocular with sides almost straight, distinctly converging toward behind. Genae pointed apically, distinctly surpassing the apex of the clypeus. Jugae angular apically. Eyes small, in lateral view approaching but not attaining the level of the lower and remote from the level of the upper surface of the head. Ratio width of eye to synthlipsis 1:1.8–2.0, 1:2.5. Antenniferous tubercles situated at or slightly before the middle of the anteocular region. First antennal segment appearing unusually short, extending only slightly beyond half the distance from its base to the apex of the head, viz., falling considerably short of the apex level of the clypeus. Second segment subcylindrical, with adpressed setae shorter than its diameter. Ratio of antennal segments 1:3.3–4.2:2.3–2.8:2.1–2.5. Rostrum slender, practically glabrous, except for a group of long hairs at the apex of the third segment (Fig. 4B). Ratio of rostral segments 1:2.2–2.8:1.1–1.2. First segment not attaining the level of the apex of antenniferous tubercles, second approaching the hind margin level of eyes. Neck dark, with a pair of light-colored spots laterally (Fig. 4C).

Pronotum very sparsely granulose, dark brown, presenting orange spots on the collar (Fig. 4D), discal callosities, on the lateral margins of the anterior lobe, submedian carinae, and on the humeral area. Anterior lobe with orange discal tubercles reduced to suboval transverse callosities, lateral tubercles absent. Posterior lobe shallowly rugose. Submedian carinae extending close to the hind margin of the sclerite. Humeri narrowly rounded, almost angular. Figure 4D shows changes in the spots and color of the pronotum. Scutellum rugose, dark brown, with a central depression. Posterior process almost entirely dark brown except in the rounded apex (Fig. 4E). Hemelytra not attaining the apex of the seventh urotergite. Corium light yellow with irregularly dark areas, the apex of the costal margin narrowly yellow. Clavus yellow with dark areas. Membrane yellowish white. Veins of membrane are narrowly dark brown; cells of membrane have a conspicuous dark spot of variable extension, centered on the vein separating cells (Fig. 4F).

**Fig. 5:**
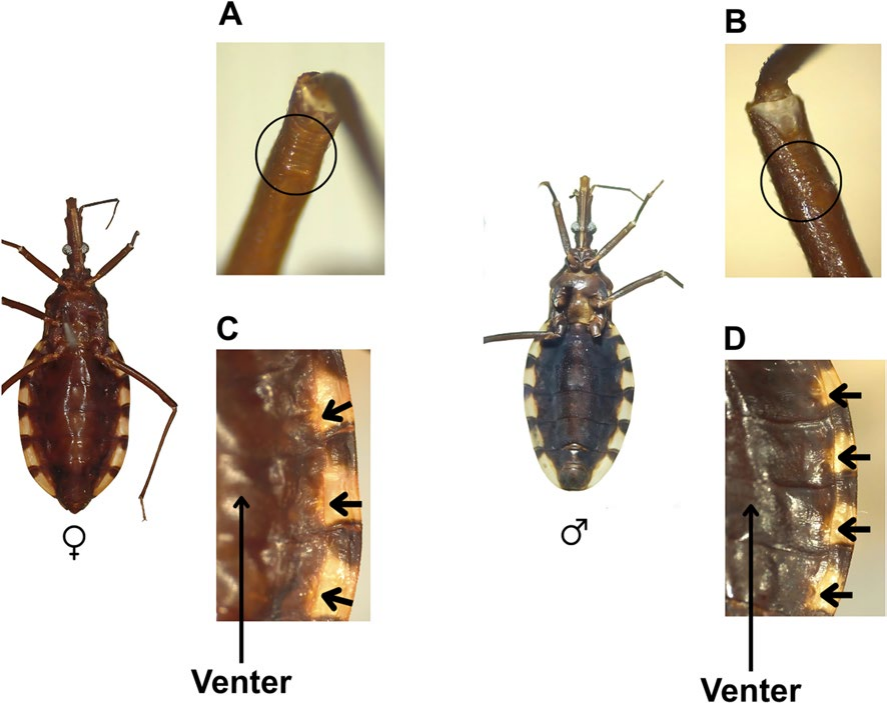
Ventral views of female and male specimens of *Triatoma chiarii* sp. nov., showing **A**, **B** denticles on the legs, and **C**, **D** position and coloration of the abdominal spiracles, as well as the coloration the connexivum. (Photographs by CD)

Legs are uniformly dark and slender. The second pair bears two inconspicuous denticles on the femora (Fig. 5A, B). Spongy fossulae absent in both sexes.

Venter delicately striated transversally, sparsely setose. Venter dark brown (Fig. 5C, D), with spiracles enclosed in small yellow areas confluent with the corresponding yellow spots of the connexivum adjoining the connexival suture, which is almost imperceptible in females (Fig. 5C). Connexivum dark brown, segments on the disc with large yellowish spot occupying two-thirds of the surface of each segment, approaching but not touching posteriorly, and distant from the anterior intersegmental suture. Both dark and light areas span the entire width of the connexival segments, with the light areas shortly extending on the respective urosternite (Fig. 5C, D). Abdominal spiracles are almost imperceptible. Table 1 summarizes the described characteristics of *T. chiarii* sp. nov. and compares them with those of *T. brasiliensis* and *T. petrocchiae*.

**Table 1:** Comparison of morphometric characteristics of *Triatoma chiarii *sp. nov., *Triatoma brasiliensis* and *Triatoma petrocchiae*

	*T. chiarii* sp. nov	*T. brasiliensis*	*T. petrocchiae*
Total length (mm)	Male 19.0–21.5; Female 19.0–24.0	Male 22.0–25.0; Female 23.0–25.5	Male 17.0–21.5; Female 18.0–23.0
Overall color	Dark brown with orange markings on the pronotum and yellow markings on the scutellum, hemelytra, and connexivum	Dark brown to black with yellow markings on the neck, pronotum, legs, hemelytra, and connexivum	Dark brown with yellow markings on the pronotum, scutellum, hemelytra, and connexivum
Head	Uniformly dark, slightly rough and granulated, slightly more than twice as long as the width of the eyes (1:0.40–0.50), and longer than the pronotum (1:0.75–0.80)	Dark brown or black, rough dorsally and laterally, distinctly longer than wide at the level of the eyes (1:0.5–0.55), and clearly longer than the pronotum (1:0.85–0.95)	Black, delicately rough and granulated, about twice as long as wide at the level of the eyes (1:0.40–0.45), and much longer than the pronotum (1:0.70–0.75)
Anteocular region	Six times longer than the postocular region (1:0.16–0.17)	Four times longer than the postocular region (1:0.25)	Four times longer than the postocular region (1:0.25)
Eyes	In lateral view, they approach but do not reach the lower level and are distant from the upper surface of the head	In lateral view, they do not reach the ventral surface and are distant from the dorsal surface	In lateral view, they do not reach the level of the ventral surface of the head and are distant from the level of the dorsal surface
First antennal article	Appears unusually short, extending only slightly beyond half the distance from its base to the apex of the head, significantly below the level of the apex of the clypeus	Reaching the level of the apex of the clypeus	Short and does not reach the level of the apex of the clypeus
Rostrum	Slim, practically glabrous, except for a group of long hairs at the apex of the third segment	Thick and as dark as the head	Narrow, almost glabrous, except for a group of long bristles at the apex of the third article
Proportions between rostral segments	1:2.2–2.8:1.1–1.2	1:1.7–1.9:0.9–1.05	1:2,2- 2,8:1,1–1,2
Neck	Dark with a pair of light spots on the sides	Dark with a pair of light lateral spots	Black with a pair of light lateral spots
Pronotum	Dark brown, with orange spots on the collar, discal callosities, on the lateral margins of the anterior lobe; Submedian carinae and in the humeral region, extending near the posterior margin of the sclerite; Anterior lobe with reduced orange discal tubercles, forming transverse suboval callosities. Lateral tubercles absent Posterior lobe slightly rugose	Dark brown to black with yellow spots on the collar; Lateral processes, discal tubercles of the anterior lobe, entire submedian carinae, and adjacent area to the carinae; Anterior lobe with very small discal tubercles, difficult to notice; Lateral tubercles absent	Dark brown collar, discal callosities, and in some cases, the lateral margins of the anterior lobe are yellowish dark brown; Anterior lobe with discal tubercles reduced to suboval callosities, and lateral tubercles absent
Scutellum	Dark brown with a central depression; Rough; Posterior process almost entirely dark brown, except for the rounded apex	Dark brown; Apical process as long as the main body of the scutellum, with a yellow tip	Dark brown; Apical process short, about three-thirds the length of the main body of the scutellum
Humeral angles	Narrowly rounded, almost angular	Rounded	Rounded, almost angular
Hemelytra	Not reaching the apex of the seventh urotergite; Pale yellow corium with irregularly dark areas, narrowly yellow at the costal margin apex; Yellow clavus with dark areas; Whitish-yellow membrane; Narrow dark brown veins on the membrane	Pale yellow corium with dark areas of variable extent; Entirely black clavus; Smoky yellow to light brown membrane; Black veins on the membrane	Reaching or almost reaching the apex of the seventh urotergite; Dark brown corium; Whitish-yellow membrane with a large central darker spot
Legs	Uniformly dark; The second pair has two inconspicuous denticles on the femurs; Sponge fossae absent in both sexes	Dark with light annulation on the femora	Black; Sponge fossae absent in both sexes

### Geometric morphometrics

#### Hemelytra

All statistical tests revealed significant differences (*P* < 0.0001) between species (Table 2). PC1 and PC2 used in the analysis of this structure accounted for a total of 71.29% of the variance (PC1 = 62.4%, PC2 = 8.89%). The species are clearly separate in the factorial maps of both the PCA and canonical correlation analysis, with no overlap. *Triatoma lenti* is the most distant species in the first, while *T. sherlocki* is the most distant in the second (Additional file 2: Figure S1; Additional file 3: Figure S2).

**Table 2:** Summary of multivariate statistical tests (multivariate ANOVA) performed in JMP software to assess morphological variation in hemelytra (wings) and heads of *Triatoma* species

	Test	Value	Approximate* F*	Number of* df*	Denominator* df*	*P* > *F*
Wings	Wilks’ lambda	0.1264105	7.6208	28	275.44	< 0.0001
	Pillai’s trace	1.3400963	5.6859	28	316	< 0.0001
	Hotelling-Lawley trace	3.5876647	9.5879	28	180.47	< 0.0001
	Roy’s max root	2.4348632	27.4792	7	79	< 0.0001
Head	Wilks’ lambda	0.0075704	20.8751	28	203.33	< 0.0001
	Pillai’s trace	2.0206758	8.6047	28	236	< 0.0001
	Hotelling-Lawley trace	26.107459	51.1335	28	130.55	< 0.0001
	Roy’s max root	22.806483	192.2261	7	59	< 0.0001

All of the tests showed statistically significant differences (*P* < 0.0001) between species for both structures

**Fig. 6A, B:**
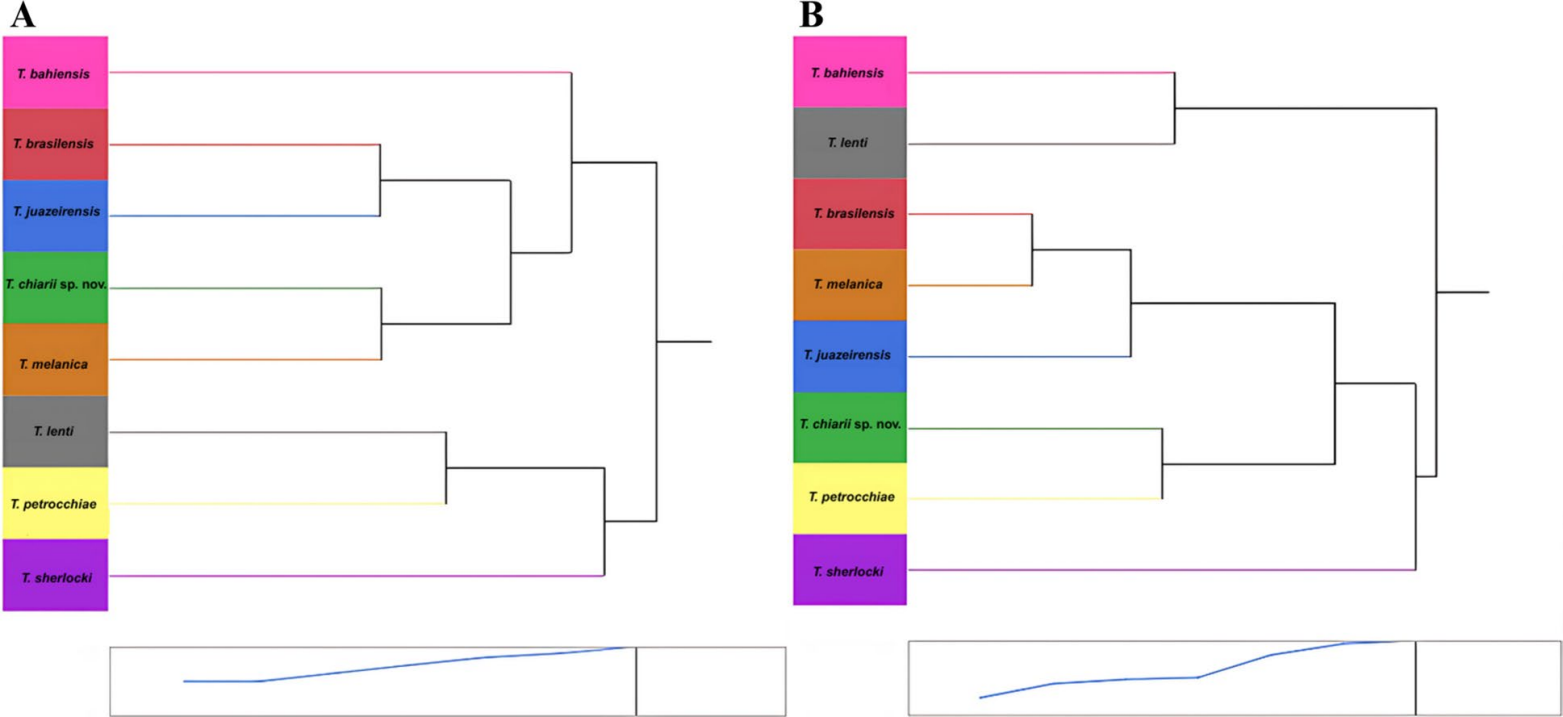
Cluster analysis of *Triatoma* species based on hemelytra and head structures. Dendograms generated from cluster analysis based on **A** hemelytra and **B** head structures of species from the *Triatoma brasiliensis* complex and *Triatoma chiarii* sp. nov

According to the clustering analysis, two major groups were observed in the dendrogram. The first comprises two subgroups: one includes *T. chiarii* sp. nov., closely related to *T. melanica*, and the other includes *T. brasiliensis* and *T. juazeirensis*, with *T. bahiensis* positioned externally to both. The second group includes *T. petrocchiae,* which is directly associated with *T. lenti*, while *T. sherlocki* appears as the outgroup species (Fig. 6A).

#### Head

As with the hemelytra analysis, all statistical tests for head shape variation revealed significant differences between the species (*P* < 0.0001; Table 2). The sum of PC1 and PC2 accounted for 80.4% of the variation (PC1 = 69.9%, PC2 = 10.5%). Once again, no overlap was observed between the species in either of the factorial maps. Two closely related groups were identified, one comprising *T. brasiliensis* with *T. juazeirensis*, *T. melanica* and *T. bahiensis*; and the other one comprising *T. petrocchiae* together with *T. chiarii* sp. nov. *Triatoma sherlocki* and *T. lenti* were identified as outgroup species (Additional file 4: Figure S3; Additional file 5: Figure S4).

The dendrogram generated by the clustering analysis revealed a major group comprising a subgroup in which *T. brasiliensis* is closely related to *T. melanica*, both in close proximity to *T. juazeirensis*. *T. chiarii* sp. nov. was directly associated with *T. petrocchiae*, while *T. sherlocki* appeared as an external species. Separately, *T. bahiensis* was directly linked to *T. lenti* (Fig. 6B).

### Phylogenetic reconstructions

**Fig. 7:**
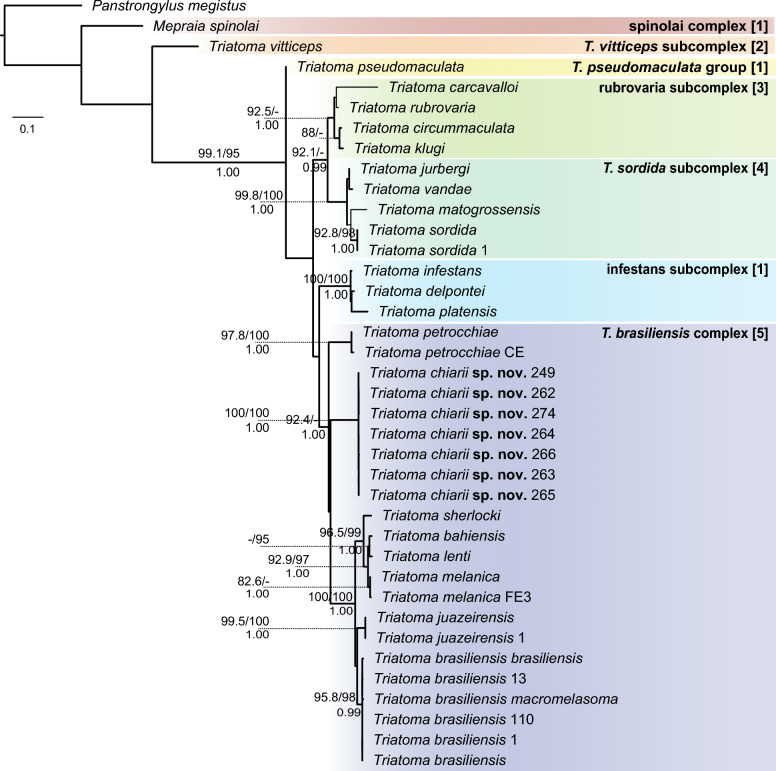
Maximum likelihood tree of triatomine species based on the concatenated molecular dataset. The tree was constructed using 428 base pairs (bp) of cytochrome b (*cytb*) and 1019 bp of the internal transcribed spacer 1 for 38 terminals representing selected groups within the South American Triatomini. Thickened branches were also recovered in Bayesian inference analysis. Clade support values are indicated as Shimodaira-Hasegawa approximate likelihood ratio test ≥ 80% / Ultrafast Bootstrap ≥ 95% above the branches, and Bayesian posterior probabilities ≥ 0.95 below. Colors indicate members of distinct groups, complexes, and subcomplexes following the classifications proposed by the following authors: Monteiro et al. [64] (*1*), Alevi et al. [65] (*2*), Schofield and Galvão [66] (*3*), Belintani et al. [51] (*4*), and Dale et al. [10] (*5*)

Phylogenetic analyses (Fig. 7; Additional file 6: Figure S5) based on concatenated datasets recovered the placement of *T. chiarii* sp. nov. within the *T. brasiliensis* species complex (SH-aLRT = 92.4, UFBoot < 95, BPP = 1.00), with *T. petrocchiae* recovered as a sister group to the remaining species of the complex. All *T. chiarii* sp. nov. samples were recovered in a strongly maximally supported clade. However, its position as sister to the clade comprising *T. brasiliensis*, *T. juazeirensis*, *T. sherlocki*, *T. bahiensis*, *T. lenti*, and *T. melanica* received weak support (SH-aLRT < 80, UFBoot < 95, BPP < 0.95). Interspecific genetic distances (K2P) between *T. chiarii* sp. nov. and other members of the *T. brasiliensis* complex were consistently high, with values ranging from 0.142 to 0.146 when compared to *T. brasiliensis*, and 0.141 to 0.147 when compared to *T. petrocchiae*. These values are markedly higher than those observed between several recognized species in the complex, such as *T. lenti* and *T. bahiensis* (0.026), *T. lenti* and *T. melanica* (0.031), or *T. brasiliensis* and *T. juazeirensis* (0.077). (Additional file 7: Table S2). Phylogenetic reconstructions based on individual markers are available in Additional file 8 (Figure S6) and Additional file 9 (Figure S7).

## Discussion

In this study, we describe a new species of triatomine within the genus* Triatoma* based on a combination of diverse analytical approaches. *Triatoma chiarii* sp. nov. morphologically exhibits a blend of traits shared with both *T. petrocchiae* and *T. brasiliensis.* Features such as the structure of the first antennal segment, a glabrous rostrum, and the absence of a spongy fossette in both sexes resemble those of *T. petrocchiae*. Conversely, characteristics typically associated with *T. brasiliensis*, including conspicuous yellow marks on the sides of the neck, a dark brown pronotum, and a scutellum with a rounded and raised posterior process, are also present [11]. However, the morphological similarity between *T. chiarii* sp. nov., *T. brasiliensis*, and *T. petrocchiae*, along with their overlapping geographic distribution, may complicate accurate identification and lead to potential misclassification of these taxa.

Morphological analyses play a crucial role in the description and validation of species [21, 40–42]. Coloration patterns in the early studies of the *T. brasiliensis* complex were particularly significant. The species currently classified by Costa et al. [21] were initially regarded as chromatic variants of *T. brasiliensis* [8, 11]. The status of *T. b. melanica* was subsequently elevated to species rank as *T. melanica* [43], *T. juazeirensis* was described [44], and *T. b. macromelasoma* was redescribed [21]. A morphological character analysis in 2016 was instrumental in revalidating *T. bahiensis* as a distinct species [45].

Although hybridization events are possible within the *T. brasiliensis* complex [46, 47], experimental crosses between *T. brasiliensis* and *T. petrocchiae* have yielded eggs with low or no viability, indicating the presence of reproductive barriers between these species [48, 49]. While reproductive compatibility has been observed between other species and subspecies within the *T. brasiliensis* complex [46, 47], further studies are needed to determine whether *T. chiarii* sp. nov. shares this capacity.

Geometric morphometrics of the wing and head in this study revealed that *T. chiarii* sp. nov. is distinctly grouped separately from *T. brasiliensis* and *T. petrocchiae*. However, it showed greater proximity to the latter, as well as to the other members of the *T. brasiliensis* species complex. Geometric morphometrics has been widely applied in various studies, focusing on both the wing [20, 26, 45, 50–52] and the head [9, 26, 53, 54], using different approaches. Using this methodology, Costa et al. [15] were the first to demonstrate homoploid hybrid speciation within the triatomine group, specifically in *T. b. macromelasoma*.

Phylogenetic reconstruction incorporating ITS-1 in addition to the widely used *cytb* marker placed all of the specimens of *T. chiarii* sp. nov. in this study within the monophyletic *T. brasiliensis* species complex with strong support. The sister group position of *T. petrocchiae* in relation to other members of the *T. brasiliensis* complex (*T. brasiliensis*, *T. juazeirensis*, *T. sherlocki*, *T. bahiensis*, *T. lenti*, and *T. melanica*) was previously documented by Oliveira et al. [9], also with high support, using multiple mitochondrial genes (16S rRNA, *COI*, *cytb*, and 12S rRNA). Notably, the phylogenetic relationships of *T. chiarii* sp. nov. in relation to the other members of the *T. brasiliensis* species complex, as well as the position of this complex in relation to other Triatomini species complexes, remain unclear, highlighting the need for additional molecular markers and broader taxonomic sampling to clarify their evolutionary history.

Genetic distance analyses based on variations in the *cytb* gene have been a key tool in delimiting species status within the Triatominae. In this context, Monteiro et al. [29] showed that distances greater than 0.075 K2P are considered sufficient to establish a newly discovered taxon as an independent evolutionary entity. Herein, we observed that all *T. chiarii* sp. nov. samples exhibited values approximately twice (0.15) the defined cutoff point.

According to Noireau et al. [55], the use of different methodologies is important for describing new species. A species definition cannot solely rely on morphology; it is essential to understand the phenotypic plasticity driven by different factors and environmental changes [56]. Geometric morphometrics has been employed in several studies as an effective tool for defining species, subspecies, and intraspecific variation within the subfamily Triatominae, with the wings and head being the most commonly used phenotypic markers to address these issues [20, 26, 39, 50, 52, 57, 58]. Likewise, molecular methods are equally critical for understanding speciation processes [29]. Thus, combining both approaches is essential for ensuring the robustness of the results [9]. Based on the results described herein, we conclude that *T. chiarii* sp. nov. is indeed a new species of triatomine and can be classified within the *T. brasiliensis* species complex.

## Conclusions

*Triatoma chiarii* sp. nov. was captured in sylvatic and peridomestic environments in regions where other triatomine species are present. *Triatoma brasiliensis*, *T. pseudomaculata*, *T. petrocchiae*, *Panstrongylus lutzi* (Neiva and Pinto, 1923), *Panstrongylus megistus* (Burmeister, 1835), *Rhodnius nasutus* Stål, 1859, and *Psammolestes tertius* Lent and Jurberg, 1965 have already been identified in the investigated areas [59–63]. The description of a new species from these areas highlights the importance of ongoing vector monitoring actions conducted by those involved in Chagas disease surveillance, as, given the high morphological similarity between other triatomine species present in the region, these actions ensure the accurate identification of triatomine species and thereby enable a timely and appropriate epidemiological response when necessary. Moreover, further investigation into other eco-epidemiological aspects related to *Triatoma chiarii* sp. nov., such as the prevalence of natural *Trypanosoma cruzi* infection, feeding sources, and its potential for infesting human dwellings, is crucial.

## Supplementary Information

Additional file 1.

Additional file 2.

Additional file 3.

Additional file 4.

Additional file 5

Additional file 6.

Additional file 7.

Additional file 8.

Additional file 9.

## Data Availability

All data generated or analyzed during this study are included in this published article and its additional files. The newly generated sequences have been deposited in the GenBank database under the accession numbers given in Additional file 1: Table S1.

## References

[1] Ferreira ILM, Silva TPT. Transmission elimination of Chagas’ disease by *Triatoma infestans* in Brazil: an historical fact. Rev Soc Bras Med Trop. 2006;39:507–9. 17160334 10.1590/s0037-86822006000500018

[2] Dias JCP, Ramos AN Jr, Gontijo ED, Luquetti A, Shikanai-Yasuda MA, Coura JR, et al. Brazilian consensus on Chagas disease, 2015. Epidemiol Serv Saúde. 2016;25:7–86. 27869914 10.5123/S1679-49742016000500002

[3] Gurgel-Gonçalves R, Galvão C, Costa J, Peterson AJ. Geographic distribution of Chagas disease vectors in Brazil based on ecological niche modeling. J Trop Med. 2012;2012:705326. 22523500 10.1155/2012/705326 PMC3317230

[4] Chagas C. Nova tripanozomiase humana. Estudos sobre a morfologia e o ciclo evolutivo de *Schizotrypanum cruzi* n. gen., n. sp. ajente etiolojico de nova entidade morbida do homem. Mem Inst Oswaldo Cruz. 1909;1:59–218.

[5] Coura JR, Junqueira ACV, Fernandes O, Valente SAS, Miles MA. Emerging Chagas disease in Amazonian Brazil. Trends Parasitol. 2002;18:171–6. 11998705 10.1016/s1471-4922(01)02200-0

[6] WHO. Chagas disease (American trypanosomiasis). 2020. https://www.who.int/chagas/disease/en/. Accessed 20 Aug 2023.

[7] Dias JCP, Machado EM, Fernandes AL, Vinhaes MC. General situation and perspectives of Chagas disease in northeastern region, Brazil. Cad Saude Publica. 2000;16:13–34. 11119317

[8] Costa J, Almeida CE, Dotson EM, Lins A, Vinhaes M, Silveira AC, et al. The epidemiologic importance of *Triatoma brasiliensis* as a Chagas disease vector in Brazil: a revision of domiciliary captures during 1993–1999. Mem Inst Oswaldo Cruz. 2003;98:443–9. 12937751 10.1590/s0074-02762003000400002

[9] Oliveira J, Marcet PL, Takiya DM, Mendonça VJ, Belintani T, Bargues MD, et al. Combined phylogenetic and morphometric information to delimit and unify the *Triatoma brasiliensis* species complex and the Brasiliensis subcomplex. Acta Trop. 2017;170:140–8. 28219669 10.1016/j.actatropica.2017.02.020 PMC8259052

[10] Dale C, Almeida CE, Mendonça VJ, Oliveira J, Rosa JA, Galvão C, et al. An updated and illustrated dichotomous key for the Chagas disease vectors of *Triatoma brasiliensis* species complex and their epidemiologic importance. Zookeys. 2018;805:33–43. 10.3897/zookeys.805.25559 PMC6300697 30588153

[11] Lent H, Wygodzinsky P. Revision of the Triatominae (Hemiptera, Reduviidae), and their significance as vectors of Chagas’ disease. Bull Amer Mus Nat Hist. 1979;163:123–520.

[12] Costa J, Barth OM, Marchon-Silva V, Almeida CE, Freitas-Sibajev MGR, Panzera F. Morphological studies on the *Triatoma brasiliensis* Neiva, 1909 (Hemiptera: Reduviidae: Triatominae) genital structure and eggs of different chromatic forms. Mem Inst Oswaldo Cruz. 1997;92:493–8.

[13] Oliveira J, Alevi KCC, Almeida CE, Mendonça VJ, Costa J, Rosa JA. *Triatoma brasiliensis* species complex: characterization of the external female genitalia. J Vector Ecol. 2020;45:57–68. 32492272 10.1111/jvec.12373

[14] Costa J, Almeida JR, Britto C, Duarte R, Marchon-Silva V, Pacheco RS. Ecotopes, natural infection and thophic resources of *Triatoma brasiliensis* (Hemiptera: Reduviidae: Triatominae). Mem Inst Oswaldo Cruz. 1998;93:7–13. 9698835 10.1590/s0074-02761998000100002

[15] Costa J, Peterson AT, Dujardin JP. Morphological evidence suggests homoploid hybridization as a possible mode of speciation in the Triatominae (Hemiptera, Heteroptera, Reduviidae). Infect Genet Evol. 2009;9:263–70. 19135177 10.1016/j.meegid.2008.12.005

[16] Borges-Pereira J, Sarquis O, Zauza PL, Britto C, Lima MM. Epidemiology of Chagas disease in four rural localities in Jaguaruana, State of Ceará. Seroprevalence of infection, parasitemia and clinical characteristics. Rev Soc Bras Med Trop. 2008;41:345–51. 18853005 10.1590/s0037-86822008000400005

[17] Almeida CE, Folly-Ramos E, Peterson AT, Lima-Neiva V, Gumiel M, Duarte R, et al. Could the bug *Triatoma sherlocki* be vectoring Chagas disease in small mining communities in Bahia, Brazil? Med Vet Entomol. 2009;23:410–7. 19941607 10.1111/j.1365-2915.2009.00822.x

[18] Almeida CE, Oliveira HL, Correia N, Dornak LL, Gumiel M, Lima-Neiva V, et al. Dispersion capacity of *Triatoma sherlocki*, *Triatoma juazeirensis* and laboratory-bred hybrids. Acta Trop. 2012;122:71–9. 22210440 10.1016/j.actatropica.2011.12.001

[19] Costa J, Dornak LL, Almeida CE, Perterson AT. Distributional potential of the *Triatoma brasiliensis* species complex at present and under scenarios of future climate conditions. Parasit Vectors. 2014;7:238. 24886587 10.1186/1756-3305-7-238 PMC4046994

[20] Kamimura EH, Viana MC, Lilioso M, Fontes FHM, Pires-Silva D, Valença-Barbosa C, et al. Drivers of molecular and morphometric variation in *Triatoma brasiliensis* (Hemiptera: Triatominae): the resolution of geometric morphometrics for populational structuring on a microgeographical scale. Parasit Vectors. 2020;13:455. 32894173 10.1186/s13071-020-04340-7 PMC7487581

[21] Costa J, Correia NC, Neiva VL, Gonçalves TCM, Felix M. Revalidation and redescription of *Triatoma brasiliensis macromelasoma* Galvão, 1956 and an identification key for the *Triatoma brasiliensis* complex (Hemiptera: Reduviidae: Triatominae). Mem Inst Oswaldo Cruz. 2013;108:785–9. 24037202 10.1590/0074-0276108062013016 PMC3970697

[22] Dujardin JP. A template-dependent semilandmarks treatment and its use in medical entomology. Infect Genet Evol. 2019;70:197–207. 30851461 10.1016/j.meegid.2019.03.002

[23] Rohlf F. Geometric morphometrics simplified. Trends Ecol Evol. 2005;20:13–4.

[24] Bookstein F. Morphometric tools for landmark data. Cambridge University Press; 1992.

[25] Coutinho CBD. Taxonomia integrada de espécies de *Triatoma* Laporte, 1832 (Hemiptera: Reduviidae: Triatominae) do Estado do Rio Grande do Sul, Brasil. https://www.arca.fiocruz.br/handle/icict/30228 (2017). Accessed 25 Nov 2024.

[26] Alvarez ACPC, Dale C, Galvão C. Geometric morphometry of the *Rhodnius prolixus* complex (Hemiptera, Triatominae): patterns of intraspecific and interspecific allometry and their taxonomic implications. ZooKeys. 2024;1202:213–28. 38826493 10.3897/zookeys.1202.108157 PMC11140263

[27] JMP Statistical Discovery LLC 2022. Using JMP® 17. Cary, NC: JMP Statistical Discovery LLC.

[28] Lyman DF, Monteiro FA, Escalante AA, Rosales CC, Wesson DM, Dujardin JP, et al. Mitochondrial DNA sequence variation among triatomine vectors of Chagas’ disease. Am J Trop Med Hyg. 1999;60:377–86. 10466963 10.4269/ajtmh.1999.60.377

[29] Monteiro FA, Donnelly MJ, Beard CB, Costa J. Nested clade and phylogeographic analyses of the Chagas disease vector *Triatoma brasiliensis* in Northeast Brazil. Mol Phylogenet Evol. 2004;32:46–56. 15186796 10.1016/j.ympev.2003.12.011

[30] Ji Y-J, Z D-X, He L-J. Evolutionary conservation and versatility of a new set of primers for amplifying the ribosomal internal transcribed spacer regions in insects and other invertebrates. Mol Ecol Notes. 2003;3:581–5.

[31] Katoh K, Standley DM. MAFFT: iterative refinement and additional methods. Methods Mol Biol. 2014;1079:131–46. 24170399 10.1007/978-1-62703-646-7_8

[32] Nguyen LT, Schmidt HA, Von Haeseler A, Minh BQ. IQ-tree: a fast and effective stochastic algorithm for estimating maximum-likelihood phylogenies. Mol Biol Evol. 2015;32:268–74. 25371430 10.1093/molbev/msu300 PMC4271533

[33] Ronquist F, Teslenko M, van der Mark P, Ayres DL, Darling A, Höhna S, et al. MrBayes 3.2: efficient Bayesian phylogenetic inference and model choice across a large model space. Syst Biol. 2012;61:539–42. 22357727 10.1093/sysbio/sys029 PMC3329765

[34] Kalyaanamoorthy S, Minh BQ, Wong TK, Von Haeseler A, Jermiin LS. ModelFinder: fast model selection for accurate phylogenetic estimates. Nat methods. 2017;14:587–9. 28481363 10.1038/nmeth.4285 PMC5453245

[35] Rambaut A, Drummond AJ. Tracer version 1.7.1. 2009. http://tree.bio.ed.ac.uk/software/tracer/. Accessed 12 Sept 2024.

[36] Guindon S, Dufayard JF, Lefort V, Anisimova M, Hordijk W, Gascuel O. New algorithms and methods to estimate maximum-likelihood phylogenies: assessing the performance of PhyML 30. Syst Biol. 2010;59:307–21. 20525638 10.1093/sysbio/syq010

[37] Minh BQ, Nguyen MAT, von Haeseler A. Ultrafast approximation for phylogenetic bootstrap. Mol Biol Evol. 2013;30:1188–95. 23418397 10.1093/molbev/mst024 PMC3670741

[38] Tamura K, Peterson D, Peterson N, Stecher G, Nei M, Kumar S. MEGA5: molecular evolutionary genetics analysis using maximum likelihood, evolutionary distance, and maximum parsimony methods. Mol Biol Evol. 2011;28:2731–9. 21546353 10.1093/molbev/msr121 PMC3203626

[39] Dorn PL, Justi SA, Dale C, Stevens L, Galvão C, Lima-Cordón R, et al. Description of *Triatoma mopan* sp. n. from a cave in Belize (Hemiptera, Reduviidae, Triatominae). ZooKeys. 2018;775:69–95. 10.3897/zookeys.775.22553 PMC6058004 30057472

[40] Rosa JA, Rocha CS, Gardim S, Pinto MC, Mendonça VJ, Ferreira Filho JCR, et al. Description of *Rhodnius montenegrensis* sp. n. (Hemiptera: Reduviidae: Triatominae) from the state of Rondônia, Brazil. Zootaxa. 2012;3478:62–76.

[41] Gonçalves TCM, Teves-Neves SC, Santos-Mallet JR, Carbajal-de-la-Fuente AL, Lopes CM. *Triatoma jatai* sp. nov. in the state of Tocantins, Brazil (Hemiptera: Reduviidae: Triatominae). Mem Inst Oswaldo Cruz. 2013;108:429–37. 23828010 10.1590/0074-0276108042013006 PMC3970630

[42] Jurberg J, Cunha V, Cailleaux S, Raigorodschi R, Lima MS, Rocha DS, et al. *Triatoma pintodiasi* sp. nov. of the *T. rubrovaria* subcomplex (Hemiptera, Reduviidae, Triatominae). Rev Pan-Amaz Saude. 2013;4:43–56.

[43] Costa J, Argolo AM, Felix M. Redescription of *Triatoma melanica* Neiva & Lent, 1941, new status (Hemiptera: Reduviidae: Triatominae). Zootaxa. 2006;1385:47–52.

[44] Costa J, Felix M. *Triatoma juazeirensis* sp. nov. from the state of Bahia, northeastern Brazil (Hemiptera: Reduviidae: Triatominae). Mem Inst Oswaldo Cruz. 2007;102:87–90. 17294006 10.1590/s0074-02762007000100015

[45] Mendonça VJ, Alevi KCC, Pinotti H, Gurgel-Goncalves R, Pita S, Guerra AL, et al. Revalidation of *Triatoma bahiensis* sherlock and serafim, 1967 (Hemiptera: Reduviidae) and phylogeny of the *T. brasiliensis* species complex. Zootaxa. 2016;4107:239–54. 27394816 10.11646/zootaxa.4107.2.6

[46] Pinotti H, Alevi KCC, Oliveira J, Ravazi A, Madeira FF, Reis YV, et al. Segregation of phenotypic characteristics in hybrids of *Triatoma brasiliensis* species complex (Hemiptera, Reduviidae, Triatominae). Infect Genet Evol. 2021;91:104798. 33676012 10.1016/j.meegid.2021.104798

[47] Correia N, Almeida CE, Lima-Neiva V, Gumiel M, Lima MM, Medeiros LMO, et al. Crossing experiments confirm *Triatoma sherlocki* as a member of the *Triatoma brasiliensis* species complex. Acta Trop. 2013;128:162–7. 23850508 10.1016/j.actatropica.2013.06.019

[48] Espínola HN. Reproductive isolation between *Triatoma brasiliensis* Neiva, 1911 and *Triatoma petrochii* Pinto & Barreto, 1925 (Hemiptera Reduviidae). Rev Bras Biol. 1971;31:277–81. 4945961

[49] Delgado LMG, Oliveira J, Ravazi A, Madeira FF, Reis YV, Pinotti H, et al. Revisiting the hybridization processes in the *Triatoma brasiliensis* complex (Hemiptera, Triatominae): reproductive isolation between *Triatoma petrocchiae* and *T. b. brasiliensis* and *T. lenti*. Insects. 2021;12:1015. 34821815 10.3390/insects12111015 PMC8621033

[50] Gurgel-Gonçalves R, Ferreira JBC, Rosa AF, Bar ME, Galvão C. Geometric morphometrics and ecological niche modelling for delimitation of near-sibling triatomine species. Med Vet Entomol. 2011;25:84–93. 21077924 10.1111/j.1365-2915.2010.00920.x

[51] Belintani T, Oliveira J, Pinotti H, Silva LA, Alevi KCC, Galvão C, et al. Phylogenetic and phenotypic relationships of the *Triatoma sordida* subcomplex (Hemiptera: Reduviidae: Triatominae). Acta Trop. 2020;212:105679. 32860747 10.1016/j.actatropica.2020.105679

[52] Paschoaletto L, Dale C, Lima-Neiva V, Carbajal-de-la-Fuente AL, Oliveira J, Benítez HÁ, et al. Morphological stasis in time? A *Triatoma**brasiliensis**brasiliensis* study using geometric morphometrics in the long run. Animals (Basel). 2022;12:1362. 35681826 10.3390/ani12111362 PMC9179344

[53] Falcone R, Ribeiro AR, Oliveira J, Mendonça VJ, Graminha M, Rosa JA. Differentiation of *Rhodnius neglectus* and *Rhodnius prolixus* (Hemiptera: Reduviidae: Triatominae) by multiple parameters. Rev Soc Bras Med Trop. 2020;53:e20190503. 32267457 10.1590/0037-8682-0503-2019 PMC7156257

[54] Zhao Y, Galvão C, Cai W. *Rhodnius micki*, a new species of Triatominae (Hemiptera, Reduviidae) from Bolivia. ZooKeys. 2021;1012:71–93. 33584109 10.3897/zookeys.1012.54779 PMC7854562

[55] Noireau F, Gutierrez T, Zegarra M, Flores R, Brenière F, Cardozo L, et al. Cryptic speciation in *Triatoma sordida* (Hemiptera: Reduviidae) from the Bolivian Chaco. Trop Med Int Health. 1998;3:364–72. 9623941 10.1046/j.1365-3156.1998.00219.x

[56] Dujardin JP, Panzera P, Schofield C. Triatominae as a model of morphological plasticity under ecological pressure. Mem Inst Oswaldo Cruz. 1999;94:223–8. 10677722 10.1590/s0074-02761999000700036

[57] Hernández ML, Abrahan LB, Dujardin JP, Gorla DE, Catalá SS. Phenotypic variability and population structure in peridomestic *Triatoma infestans* (Hemiptera, Reduviidae): influence of macro and micro habitat. Vector Borne Zoonotic Dis. 2011;11:503–13. 20925525 10.1089/vbz.2009.0253

[58] Dujardin JP. Modern morphometrics of medically important insects. In: Tibayrenc M, editor. Amsterdam: Elsevier. Genet Evol Infect Dis. 2011:473–501.

[59] Barbosa-Silva AN, Câmara ACJ, Martins K, Nunes DF, Oliveira PIC, Azevedo PRM, et al. Characteristics of triatomine infestation and natural *Trypanosoma cruzi* infection in the state of Rio Grande do Norte, Brazil. Rev Soc Bras Med Trop. 2016;49:57–67. 27163565 10.1590/0037-8682-0300-2015

[60] Barbosa-Silva AN, Diotaiuti L, Câmara ACJ, Oliveira PIC, Galvão LMC, Chiari E, et al. First report of *Psammolestes tertius* Lent & Jurberg, 1965 (Hemiptera, Reduviidae, Triatominae) in Rio Grande do Norte state, Brazil. Check List. 2018;14:1109–13.

[61] Barbosa-Silva AN, Souza RCM, Diotaiuti L, Aguiar LMA, Câmara ACJ, et al. Synanthropic triatomines (Hemiptera: Reduviidae): infestation, colonization, and natural infection by trypanosomatids in the state of Rio Grande do Norte, Brazil. Rev Soc Bras Med Trop. 2019;52:e20190061. 31340365 10.1590/0037-8682-0061-2019

[62] Lima-Oliveira TM, Fontes FHM, Lilioso M, Pires-Silva D, Teixeira MMG, Meza JGV, et al. Molecular eco-epidemiology on the sympatric Chagas disease vectors *Triatoma brasiliensis* and *Triatoma petrocchiae*: Ecotopes, genetic variation, natural infection prevalence by trypanosomatids and parasite genotyping. Acta Trop. 2020;201:1051. 10.1016/j.actatropica.2019.105188 31545949

[63] Honorato NRM, Barbosa-Silva AN, Negreiros CCA, Aguiar LMA, Marliére NP, Souza RCM, et al. Triatomine and *Trypanosoma cruzi* discrete typing units distribution in a semi-arid area of northeastern Brazil. Acta Trop. 2021;220:105950. 33979639 10.1016/j.actatropica.2021.105950

[64] Monteiro FA, Weirauch C, Felix M, Lazoski C, Abad-Franch F. Evolution, systematics, and biogeography of the triatominae, vectors of Chagas disease. Adv Parasitol. 2018;99:265–344. 29530308 10.1016/bs.apar.2017.12.002

[65] Alevi KCC, Oliveira J, Rocha DS, Galvão C. Trends in taxonomy of Chagas disease vectors (Hemiptera, Reduviidae, Triatominae): from Linnaean to integrative taxonomy. Pathogens. 2021;10:1627. 34959582 10.3390/pathogens10121627 PMC8706908

[66] Schofiel CJ, Galvão C. Classification, evolution, and species groups within the Triatominae. Acta Trop. 2009;110:88–100. 19385053 10.1016/j.actatropica.2009.01.010

