# Supplementary material for: Triatoma chiarii sp. nov. (Hemiptera, Reduviidae, Triatominae): a new species in the Triatoma brasiliensis complex from Rio Grande do Norte state, Brazil
Source: Parasit Vectors. 2025 Dec 1;19:9. doi: 10.1186/s13071-025-07014-4 (PMC12771862; doi:10.1186/s13071-025-07014-4)
Supplement: Supplementary file 1 — Additional file 1. [file 13071_2025_7014_MOESM1_ESM.docx]

Table S1 Species included in the phylogenetic analysis and their corresponding GenBank accession numbers

| **Species** | ***Cyt b*** | **ITS-1** |
| --- | --- | --- |
| *Triatoma brasiliensis* 1*** | PV604429 | - |
| *Triatoma brasiliensis* 13* | PV604430 | PV578823 |
| *Triatoma brasiliensis* 110* | PV604431 | PV578824 |
| *Triatoma chiarii* sp. nov. (249)* | - | PV578825 |
| *Triatoma chiarii* sp. nov. (262)* | PV604432 | PV578826 |
| *Triatoma chiarii* sp. nov. (263)* | PV604433 | PV578827 |
| *Triatoma chiarii* sp. nov. (264)* | PV604434 | PV578828 |
| *Triatoma chiarii* sp. nov. (265)* | PV604435 | PV578829 |
| *Triatoma chiarii* sp. nov. (266)* | PV604436 | PV578830 |
| *Triatoma chiarii* sp. nov. (274)* | PV604437 | - |
| *Triatoma juazeirensis* 1*** | PV604438 | - |
| *Triatoma melanica* FE3*** | PV604439 | - |
| *Triatoma petrocchiae* CE*** | PV604440 | - |
| *Triatoma sordida* 1*** | PV604441 | - |
| *Triatoma bahiensis* | KT347298 | - |
| *Triatoma lenti* | KT347299 | - |
| *Triatoma brasiliensis* | KT336389 | KJ125140 |
| *Triatoma carcavalloi* | KC249244 | MW445905 |
| *Triatoma circummaculata* | MZ383988 | MW445904 |
| *Triatoma delpontei* | KC249248 | AJ576060 |
| *Triatoma infestans* | KC249257 | AJ576052 |
| *Triatoma juazeirensis* | AY494168 | KJ125151 |
| *Triatoma jurbergi* | KC249264 | - |
| *Triatoma klugi* | KC249265 | - |
| *Triatoma matogrossensis* | KC249272 | - |
| *Triatoma melanica* | AY336527 | KJ125147 |
| *Triatoma petrocchiae* | KY654075 | - |
| *Triatoma platenses* | KC249274 | AJ576061 |
| *Triatoma pseudomaculata* | KC249277 | - |
| *Triatoma rubrovaria* | KC249281 | KJ125149 |
| *Triatoma sherlocki* | KC249288 | KJ125149 |
| *Triatoma sordida* | MH054941 | AJ576063 |
| *Triatoma vandae* | KC249300 | - |
| *Triatoma vitticeps* | AJ576054 | - |
| *Mepraia spinolai* | JN102358 | - |
| *Triatoma b. macromelasoma* | - | KJ125144 |
| *Panstrongylus megistus* | KC249228 | HF678465 |

*Triatomine samples sequenced in this study
