# Supplementary figures and images for: Triatoma chiarii sp. nov. (Hemiptera, Reduviidae, Triatominae): a new species in the Triatoma brasiliensis complex from Rio Grande do Norte state, Brazil
Source: Parasit Vectors. 2025 Dec 1;19:9. doi: 10.1186/s13071-025-07014-4 (PMC12771862; doi:10.1186/s13071-025-07014-4)

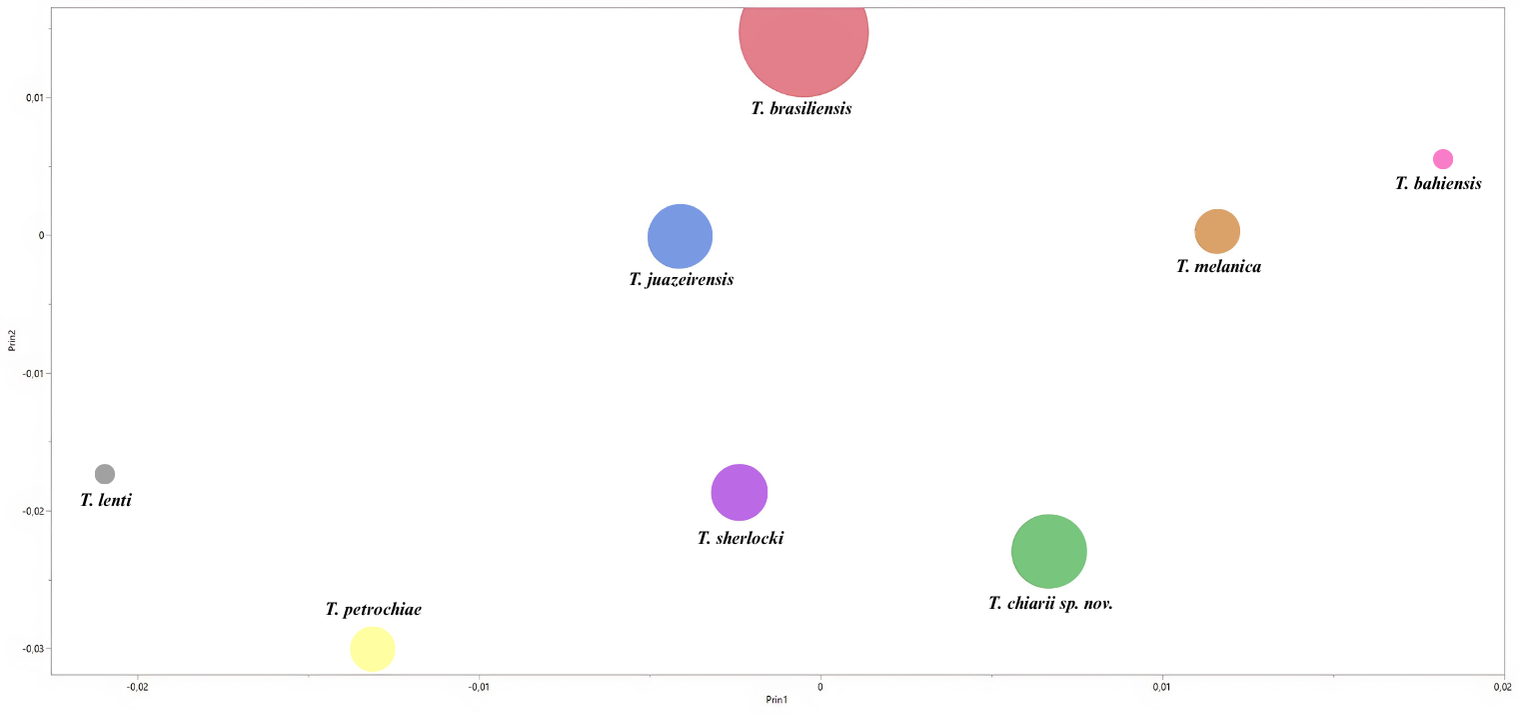

Supplement: Supplementary file 2 — Additional file 2. [file 13071_2025_7014_MOESM2_ESM.tiff]

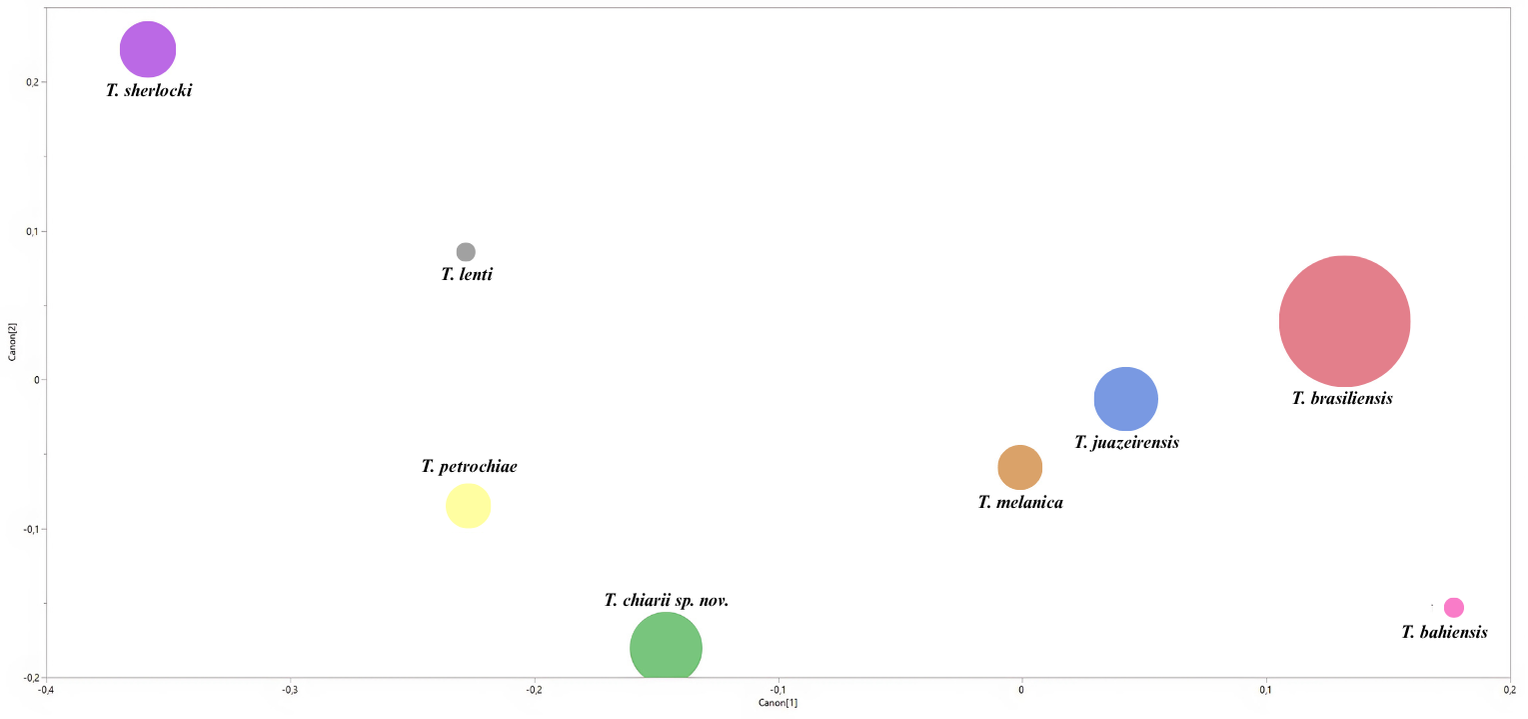

Supplement: Supplementary file 3 — Additional file 3. [file 13071_2025_7014_MOESM3_ESM.tiff]

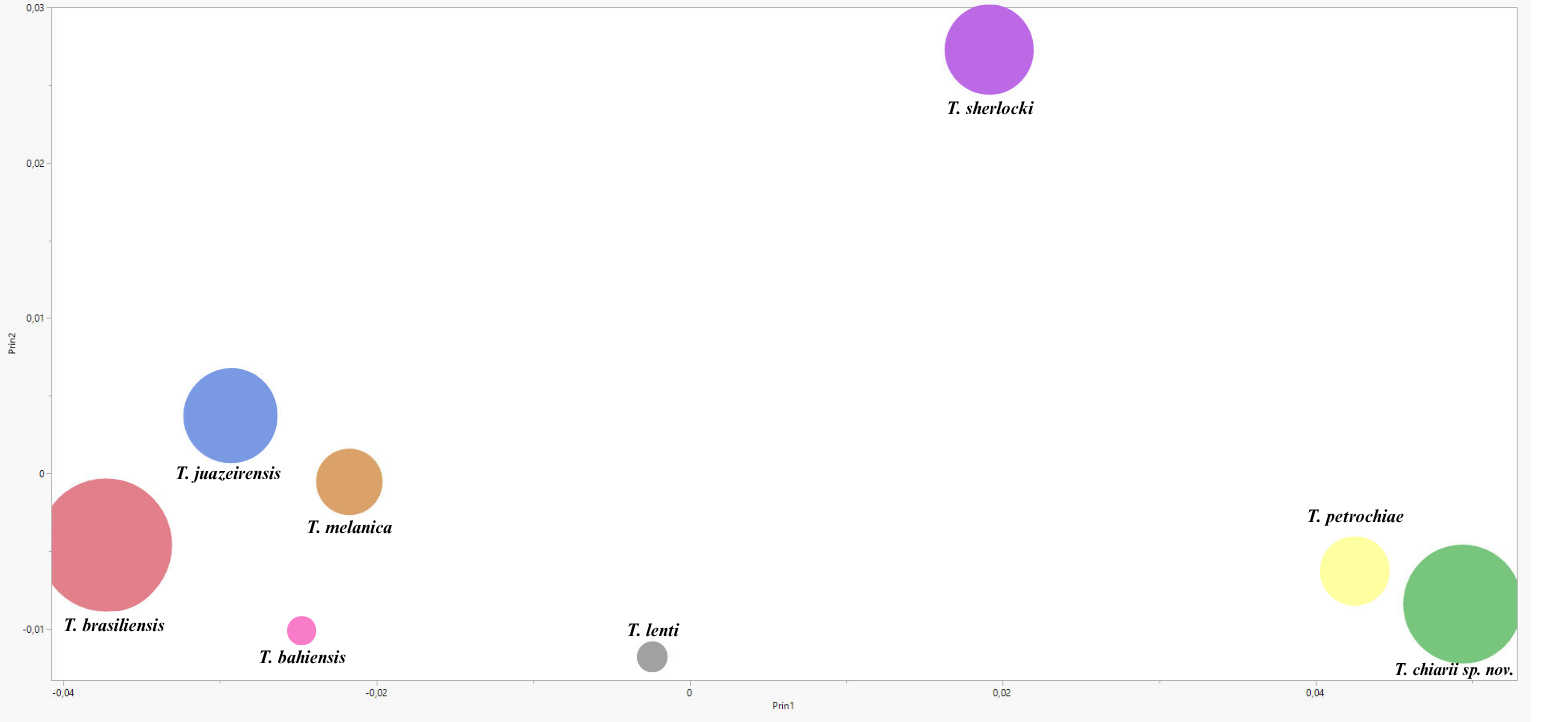

Supplement: Supplementary file 4 — Additional file 4. [file 13071_2025_7014_MOESM4_ESM.tiff]

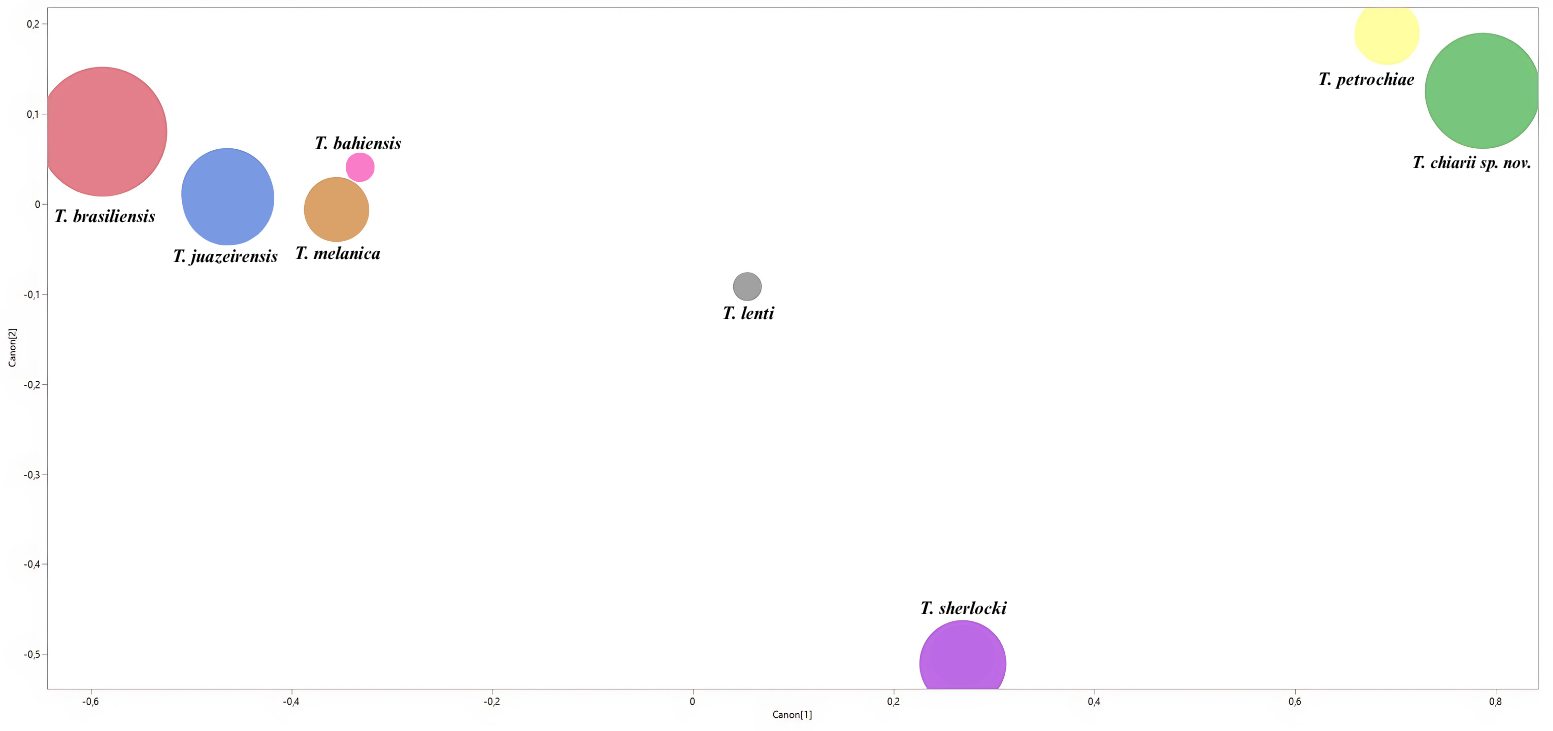

Supplement: Supplementary file 5 — Additional file 5 [file 13071_2025_7014_MOESM5_ESM.tiff]

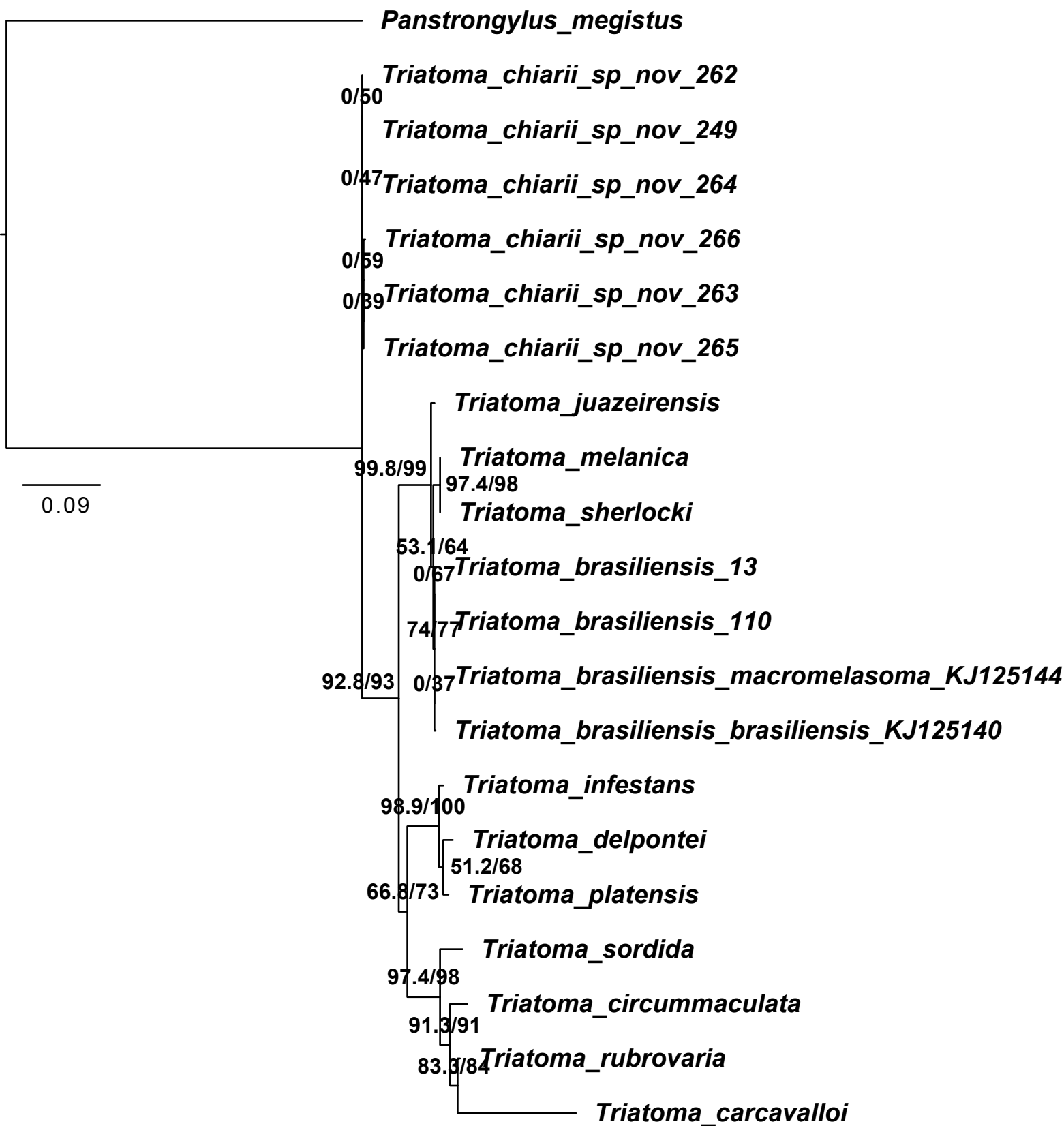

Supplement: Supplementary file 6 — Additional file 6. [file 13071_2025_7014_MOESM6_ESM.pdf]

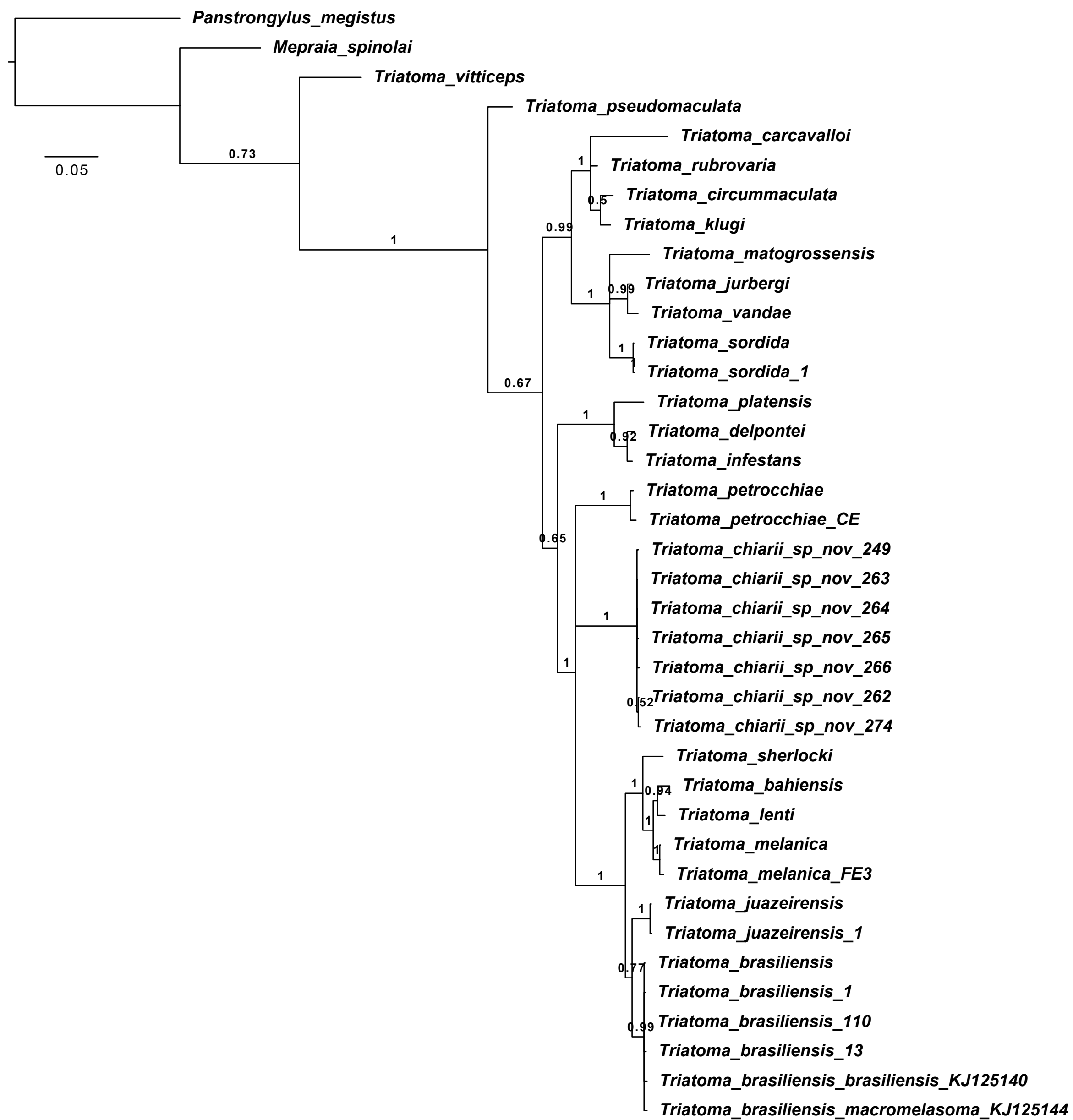

Supplement: Supplementary file 8 — Additional file 8. [file 13071_2025_7014_MOESM8_ESM.pdf]

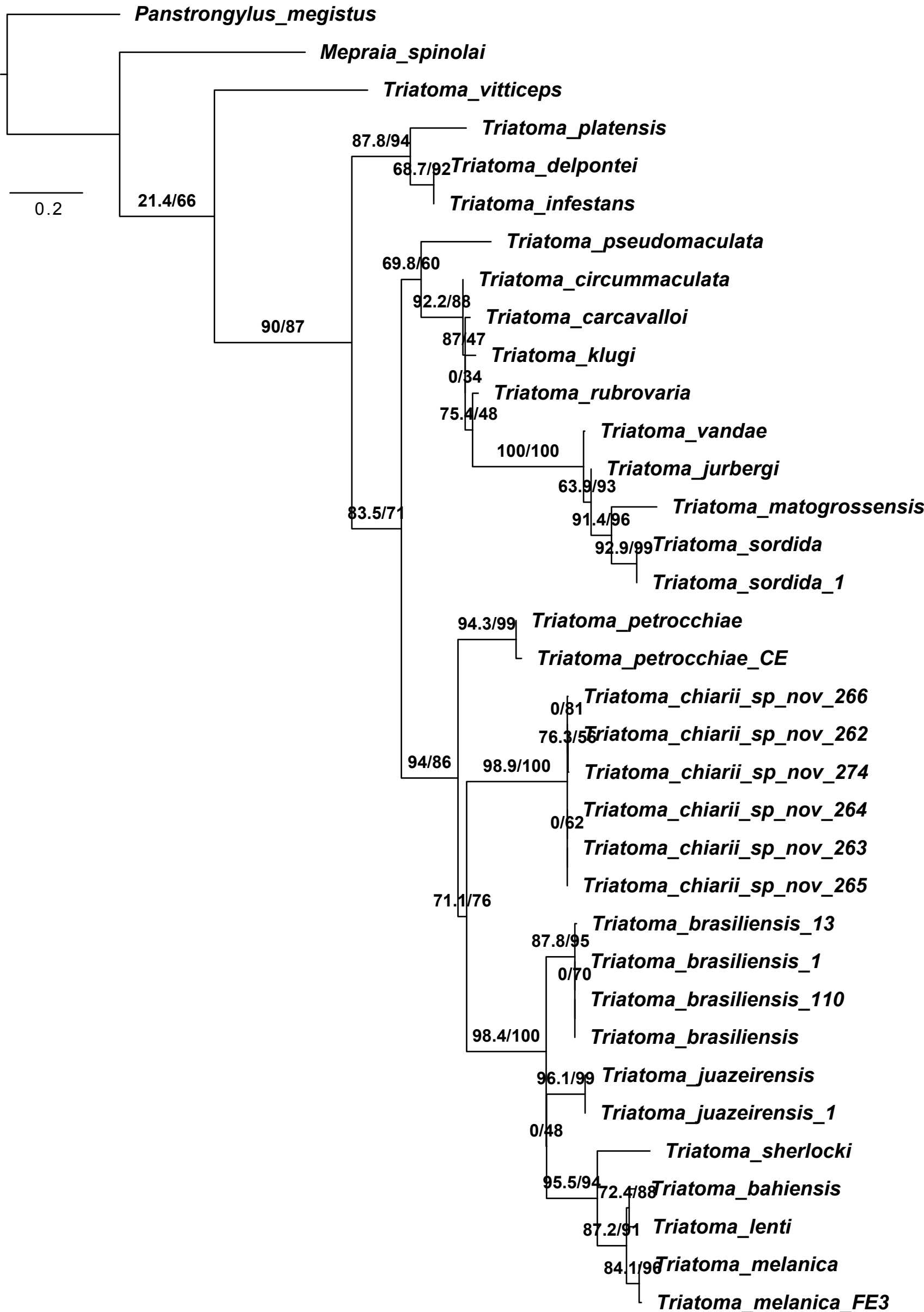

Supplement: Supplementary file 9 — Additional file 9. [file 13071_2025_7014_MOESM9_ESM.pdf]
